# Supplementary material for: Metastatic Melanoma Progression Is Associated with Endothelial Nitric Oxide Synthase Uncoupling Induced by Loss of eNOS:BH4 Stoichiometry
Source: Int J Mol Sci. 2021 Sep 3;22(17):9556. doi: 10.3390/ijms22179556 (PMC8430733; doi:10.3390/ijms22179556)
Supplement: Supplementary file 1 [file ijms-22-09556-s001.zip › ijms-1296836-supplementary.pdf]

Supplementary Materials

Metastatic melanoma progression is associated with endothelial nitric oxide synthase uncoupling induced by loss of eNOS:BH4 stoichiometry

Fabiana Henriques Machado de Melo[1,2*], Diego Assis Gonçalves[3,4], Ricardo Xisto de Sousa[5], Marcelo Yudi Icimoto[6], Denise C Fernandes[7], Francisco Laurindo[7], Miriam Galvonas Jasiulionis[1*].

* Correspondence:

FHMM: fabihmmelo2020@gmail.com, fabihmmelo@usp.br

MGJ: mjasiulionis@gmail.com, mgjasiulionis@unifesp.br

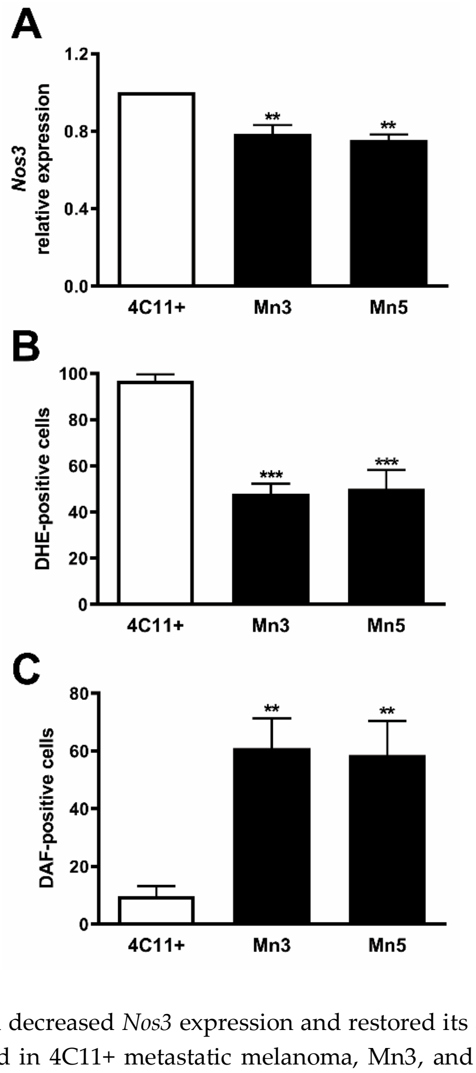

Figure S1. Superoxide depletion decreased *Nos3* expression and restored its activity. **(A)** Relative endothelial nitric oxide synthase mRNA expression was analyzed in 4C11+ metastatic melanoma, Mn3, and Mn5 derived-melan-a cells by real-time qPCR using specific primers. **(B)** Superoxide anion levels were analyzed using DHE and **(C)** nitric oxide amount were evaluated using DAF by flow cytometry. Values are reported in the bar graphs and expressed as the means ± S.D. The experiments were performed in triplicate and p values were based on the One-Way ANOVA test followed by Bonferroni post-test *p<0.05,**p<0.01; ***p<0.001.

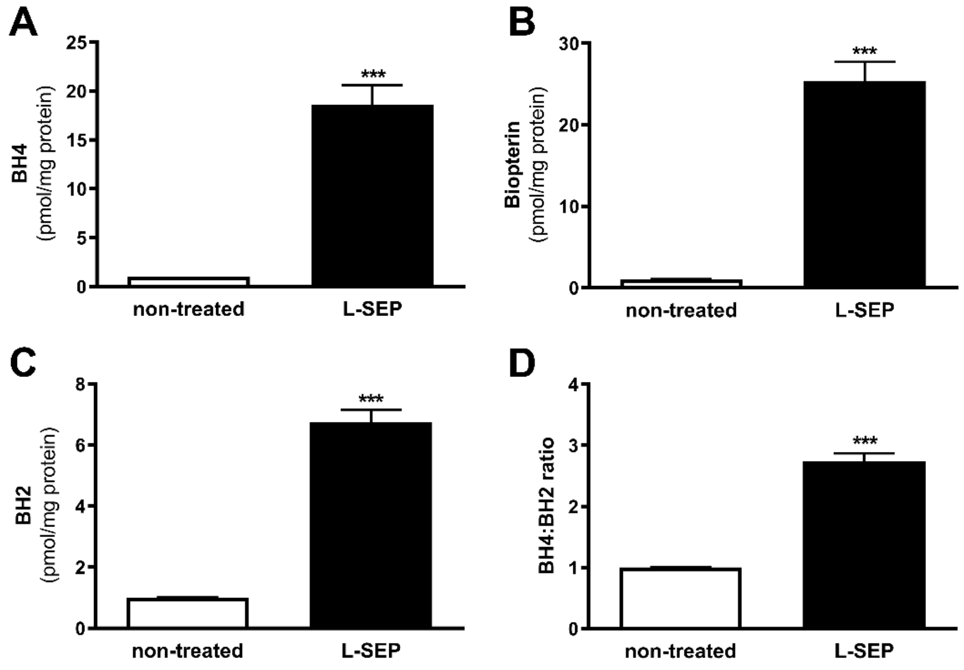

Figure S2. L-sepiapterin treatment increased tetrahydrobiopterin concentration in metastatic melanoma cells. **(A)** The amount of BH4; **(B)** BH2, **(C)** BH4:BH2 ratio and **(D)** total biopterin of 4C11+ metastatic melanoma cells treated or not (non-treated) with 40μM L-sepiapterin (L-SEP) were determined by HPLC. Values are reported in the bar graphs and expressed as the means ± S.D. The experiments were performed in triplicate and p values were based on Students' t-test ***$p<0.001$.

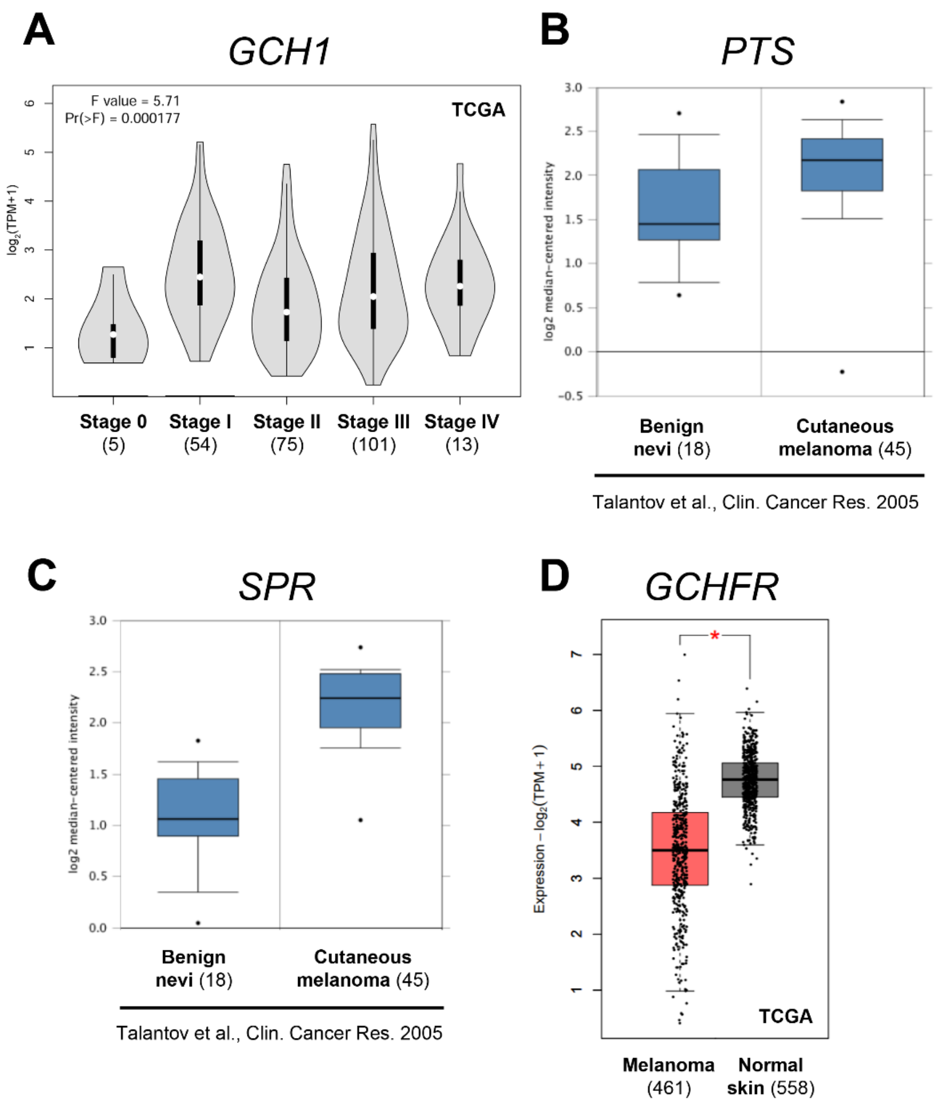

Figure S3. Expression of enzymes from BH4 *de novo* synthesis pathway in human melanoma compared with benign nevi and normal skin. **(A)** Increased expression of *GCH1* along with melanoma progression (TCGA) (n = 248). **(B)** Cutaneous melanoma (n = 45) express higher levels of *PTS* and **(C)** *SPR* compared to benign nevi (n = 18) (GSE3189). Boxes represent the interquartile range (25th–75th percentile). Microarray data by the Talantov study was obtained from the Oncomine database as indicated. The bars denote the median. P <-1E5. **(D)** Melanoma (n = 461) express lower levels of *GCHFP* compared to normal skin (n = 558). Data regarding melanoma progression was obtained from gepia2 (http://www.gepia2.cancer-pku.cn/#analysis).
